# Supplementary material for: Sex-specific splicing occurs genome-wide during early Drosophila embryogenesis
Source: eLife. 2023 Jul 19;12:e87865. doi: 10.7554/eLife.87865 (PMC10400075; doi:10.7554/eLife.87865)
Supplement: Figure 1—source data 1. [file elife-87865-fig1-data1.docx]

| **Sex** | **Stage** | **Total**  **Exon** | **Constitutive exon** | **Alternatively Spliced Exons** | | | | | | | |
| --- | --- | --- | --- | --- | --- | --- | --- | --- | --- | --- | --- |
|  |  |  |  | **Total** | **SE** | **A5SS** | **A3SS** | **MXE** | **RI** | **AF** | **AL** |
| Female | 0-2 Hr Embryo | 66927 | 56036 | 10891 | 1703 | 1918 | 1701 | 1519 | 1060 | 2626 | 364 |
| Male | 0-2 Hr Embryo | 66927 | 56296 | 10631 | 1708 | 1923 | 1692 | 1293 | 1060 | 2591 | 364 |
| Female | 2-4 Hr Embryo | 66927 | 54597 | 12330 | 1874 | 2075 | 1866 | 2018 | 1177 | 2937 | 383 |
| Male | 2-4 Hr Embryo | 66927 | 54831 | 12096 | 1841 | 2022 | 1839 | 2008 | 1146 | 2868 | 372 |
