# Supplementary figures and images for: Sex-specific splicing occurs genome-wide during early Drosophila embryogenesis
Source: eLife. 2023 Jul 19;12:e87865. doi: 10.7554/eLife.87865 (PMC10400075; doi:10.7554/eLife.87865)

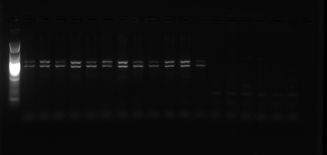

Supplement: Figure 2—figure supplement 3—source data 1. [file elife-87865-fig2-figsupp3-data1.zip › Figure 2-figure supplement 3D Source data 1 _original]

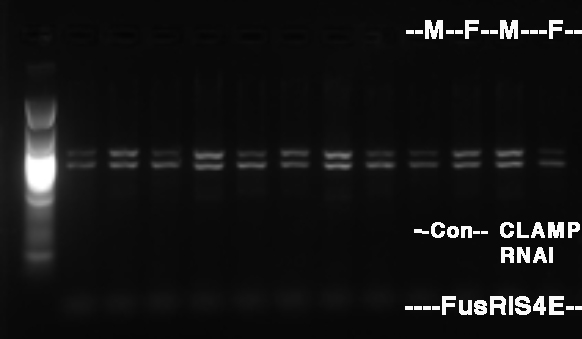

Supplement: Figure 2—figure supplement 3—source data 1. [file elife-87865-fig2-figsupp3-data1.zip › Figure 2-figure supplement 3D Source data 1 _Fus]

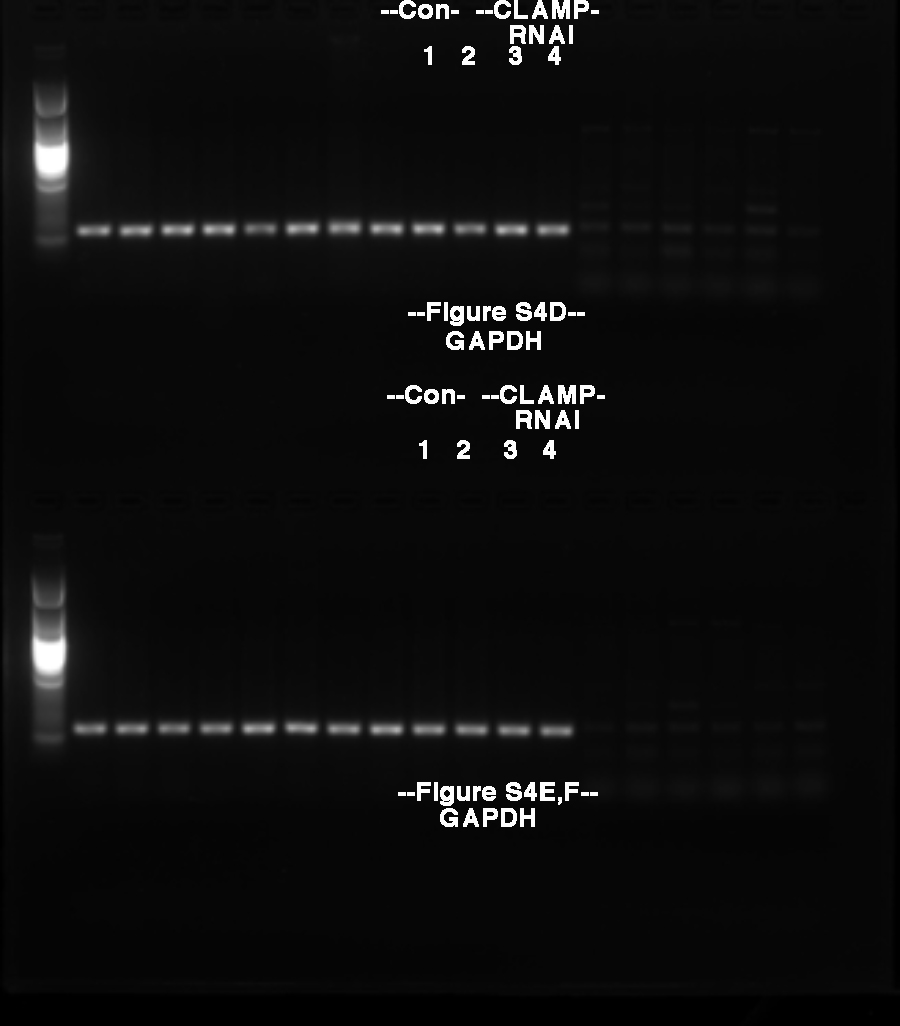

Supplement: Figure 2—figure supplement 3—source data 2. [file elife-87865-fig2-figsupp3-data2.zip › Figure 2-figure supplement 3D,E,F Source data 2 _GAPDH]

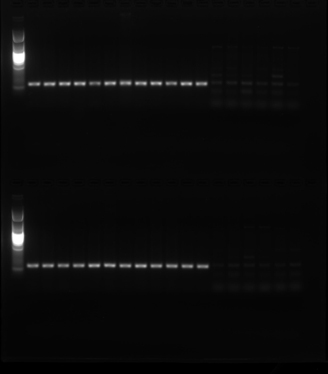

Supplement: Figure 2—figure supplement 3—source data 2. [file elife-87865-fig2-figsupp3-data2.zip › Figure 2-figure supplement 3D,E,F Source data 2_original]

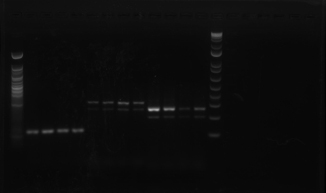

Supplement: Figure 2—figure supplement 3—source data 3. [file elife-87865-fig2-figsupp3-data3.zip › Figure 2-figure supplement 3E Source data 3 _original]

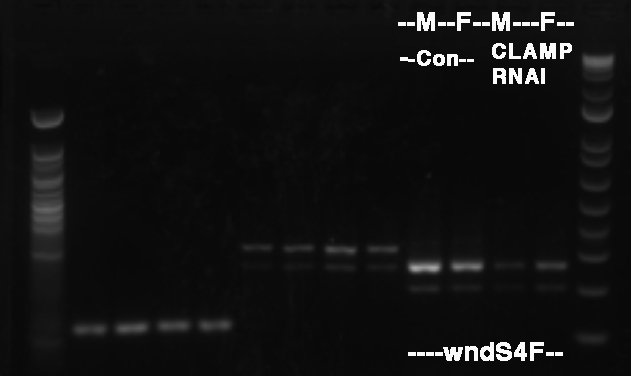

Supplement: Figure 2—figure supplement 3—source data 3. [file elife-87865-fig2-figsupp3-data3.zip › Figure 2-figure supplement 3E Source data 3 _wnd]

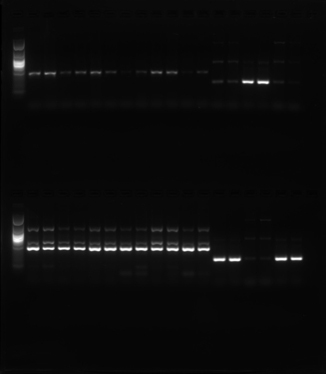

Supplement: Figure 2—figure supplement 3—source data 4. [file elife-87865-fig2-figsupp3-data4.zip › Figure 2-figure supplement 3F Source data 4 _original]

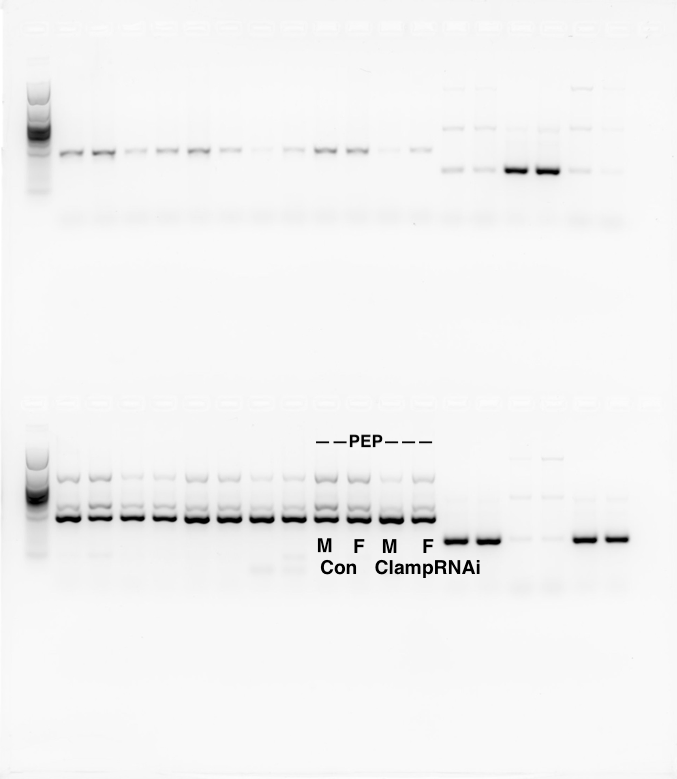

Supplement: Figure 2—figure supplement 3—source data 4. [file elife-87865-fig2-figsupp3-data4.zip › Figure 2-figure supplement 3F Source data 4 _PEP.png]

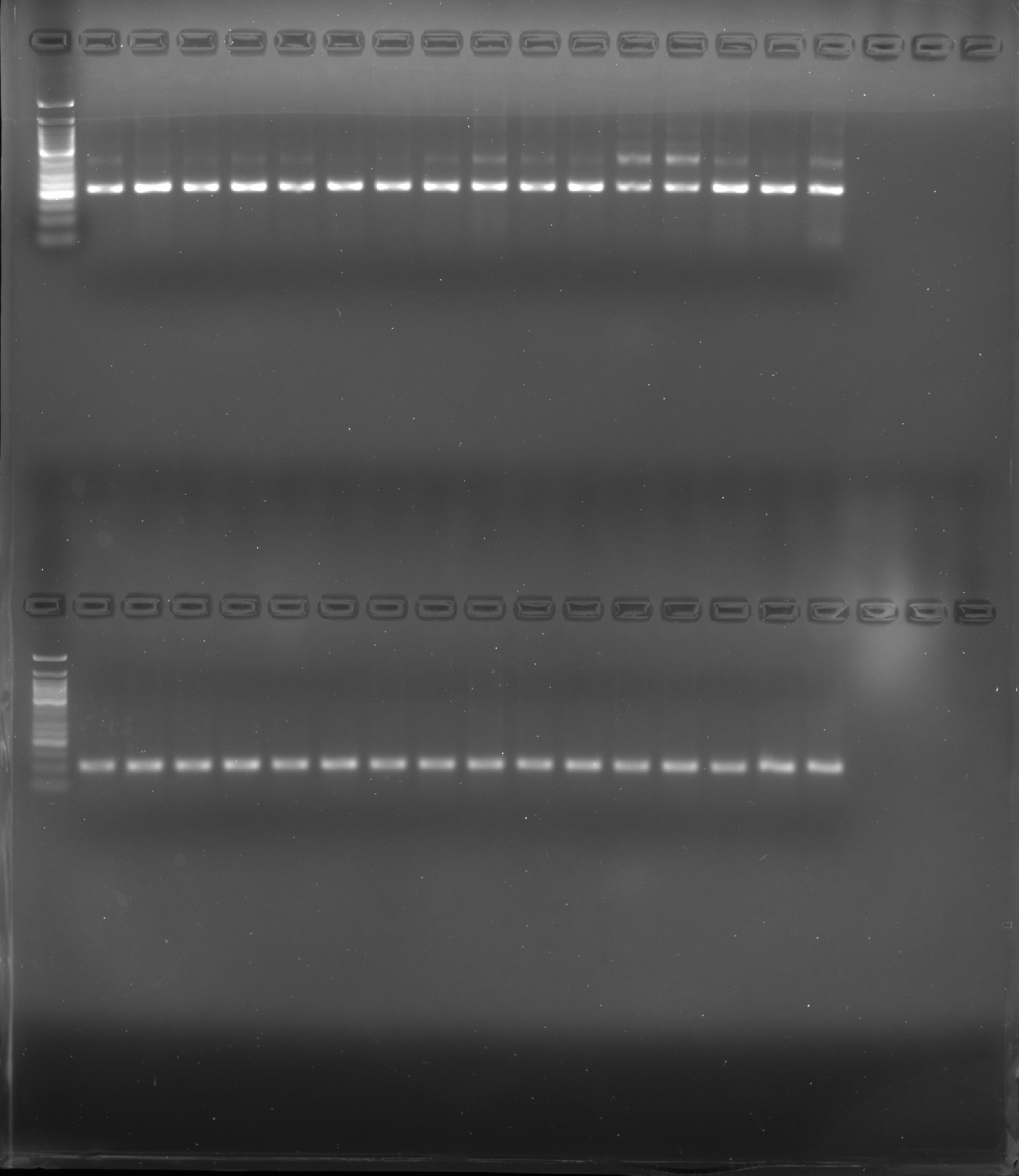

Supplement: Figure 5—source data 1. [file elife-87865-fig5-data1.zip › Figure 5A Source data 1_original.jpg]

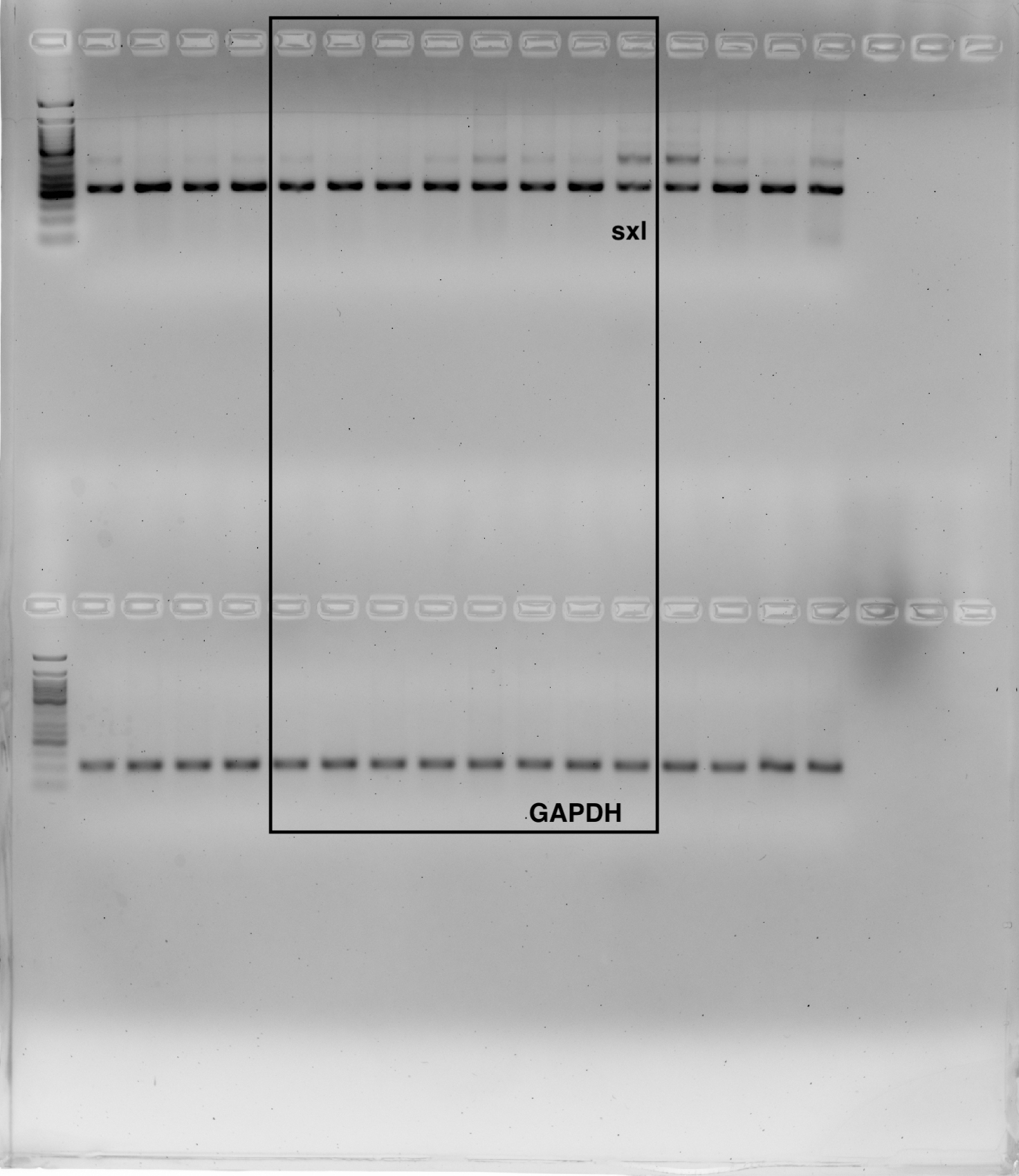

Supplement: Figure 5—source data 1. [file elife-87865-fig5-data1.zip › Figure 5A Source data 1 _sxl and GAPDH.jpeg]

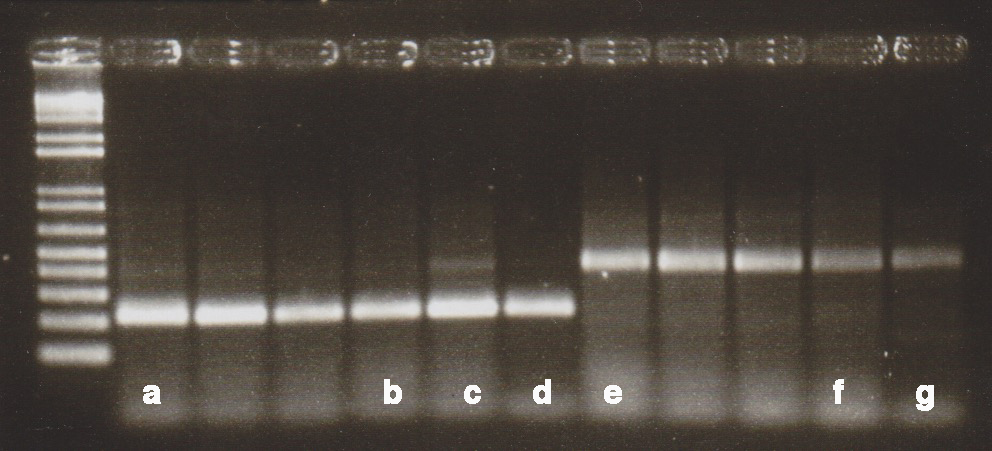

Supplement: Figure 5—source data 2. [file elife-87865-fig5-data2.zip › Figure 5B Source data 2]

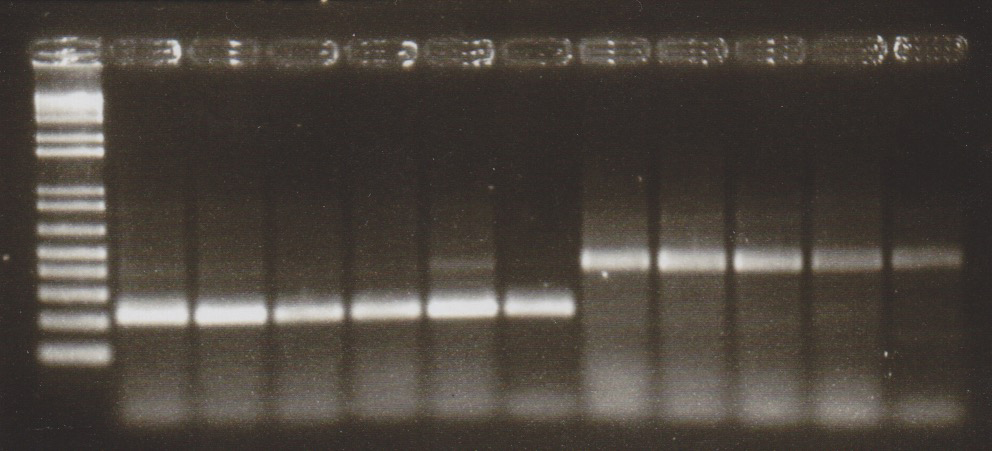

Supplement: Figure 5—source data 2. [file elife-87865-fig5-data2.zip › Figure 5B Source data 2_original]

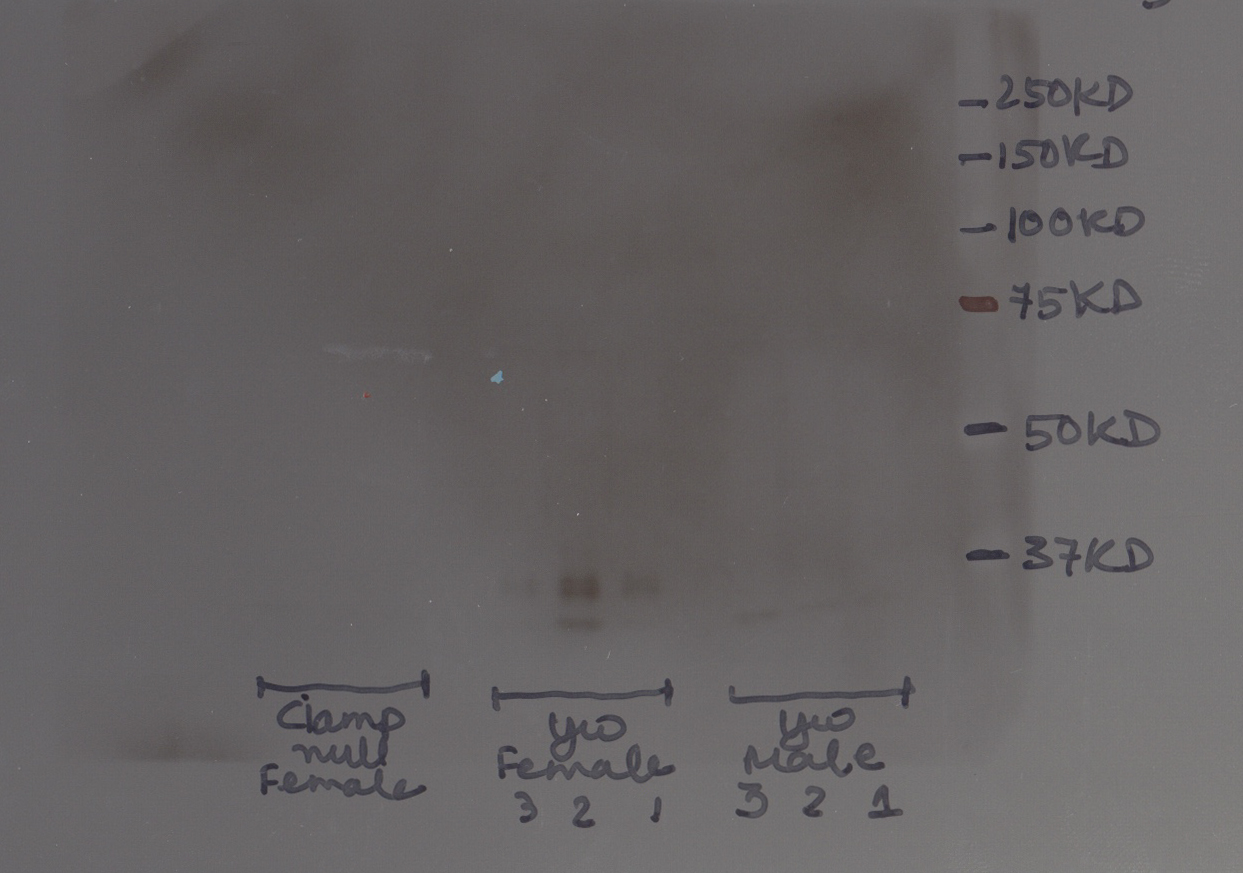

Supplement: Figure 5—source data 3. [file elife-87865-fig5-data3.zip › Figure 5D Source data 3_original]

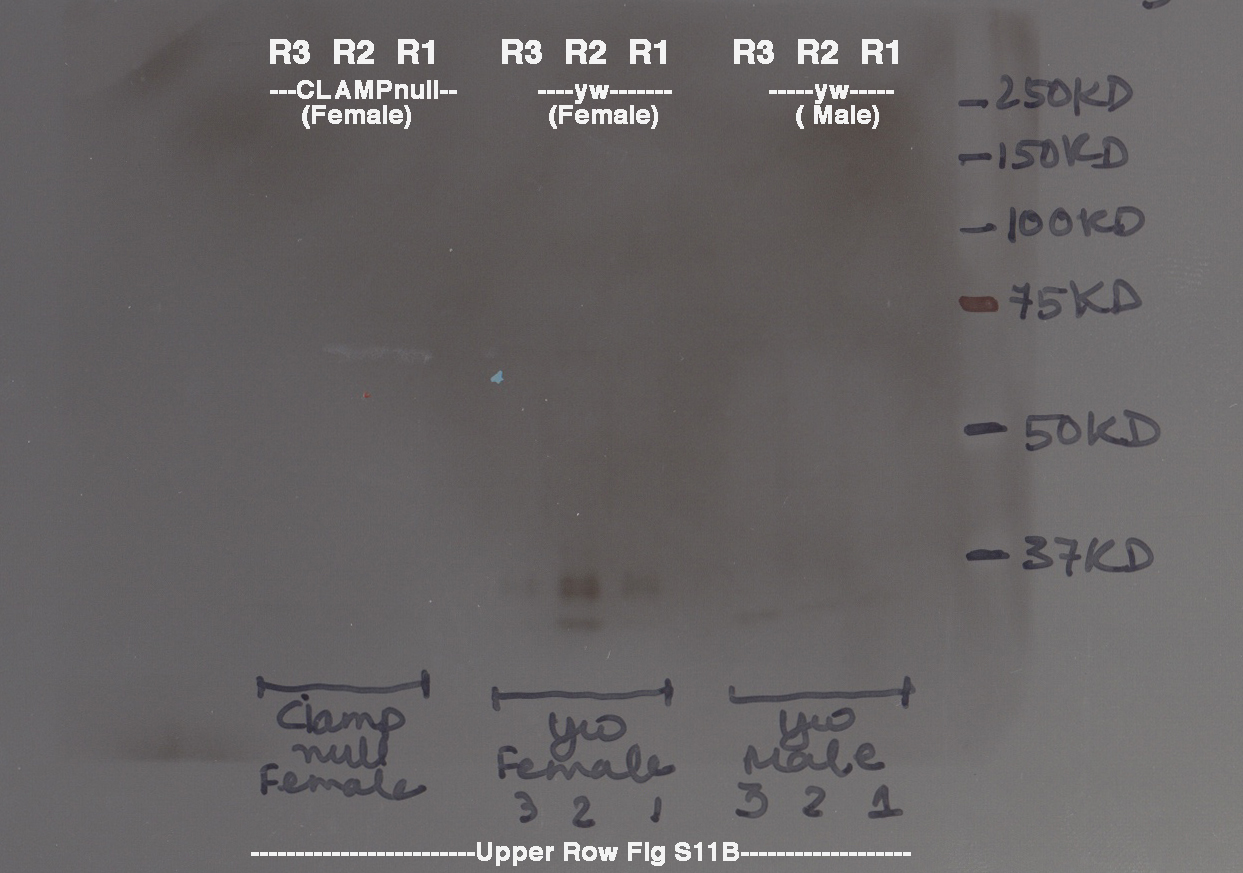

Supplement: Figure 5—source data 3. [file elife-87865-fig5-data3.zip › Figure 5D Source data 3_Sxl]

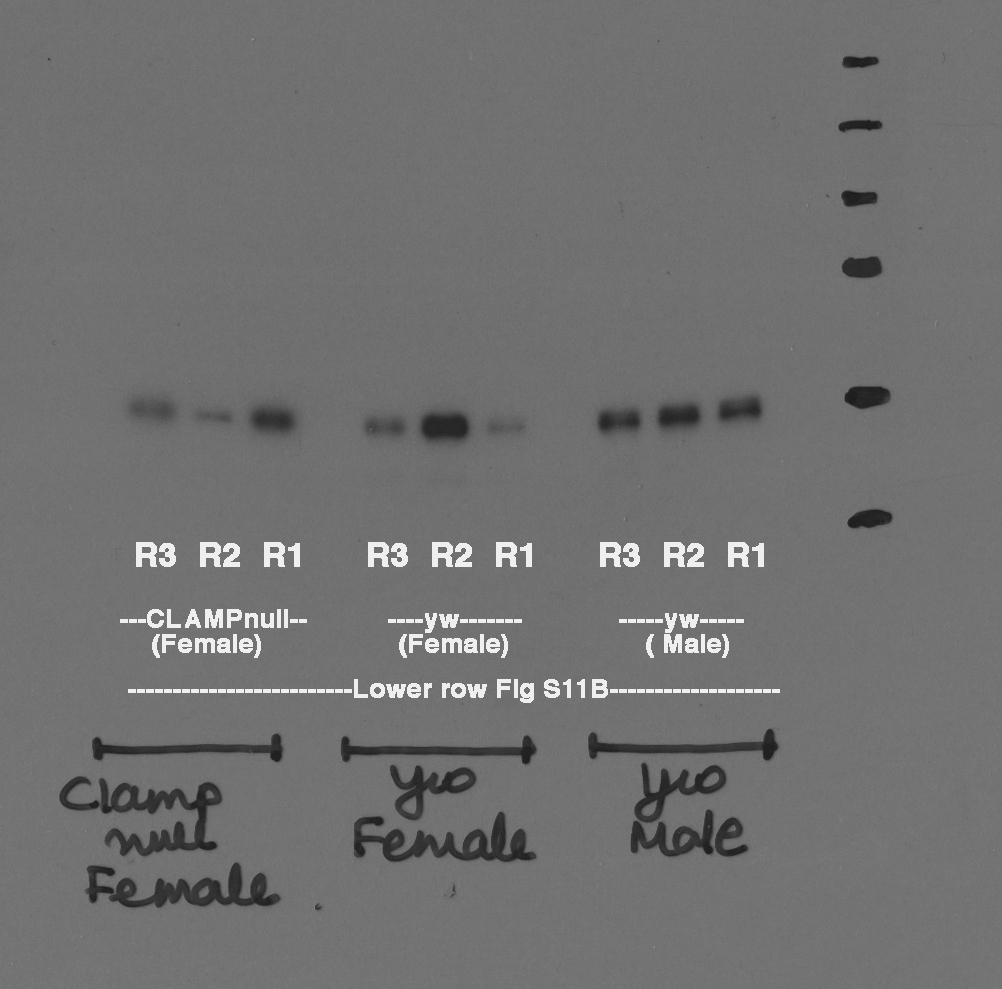

Supplement: Figure 5—source data 4. [file elife-87865-fig5-data4.zip › Figure 5D Source data 4_Tubulin]

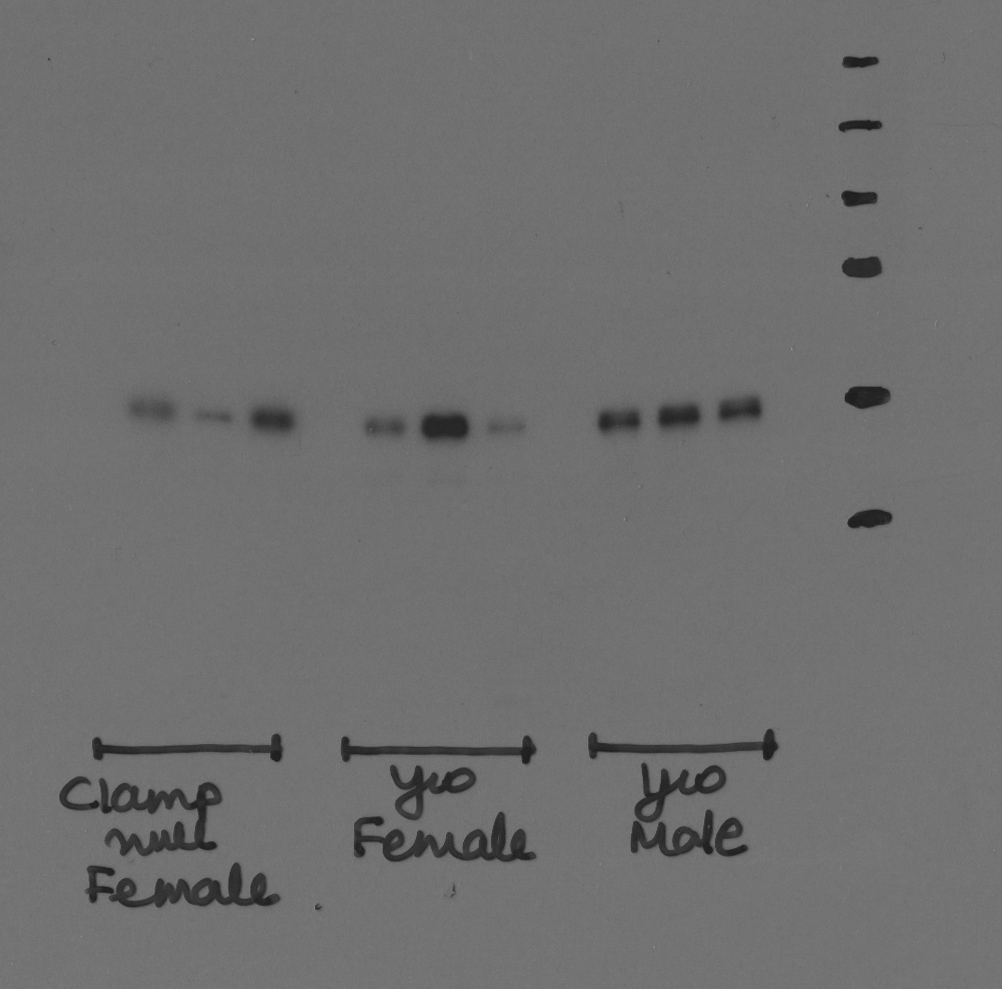

Supplement: Figure 5—source data 4. [file elife-87865-fig5-data4.zip › Figure 5D Source data 4_original]

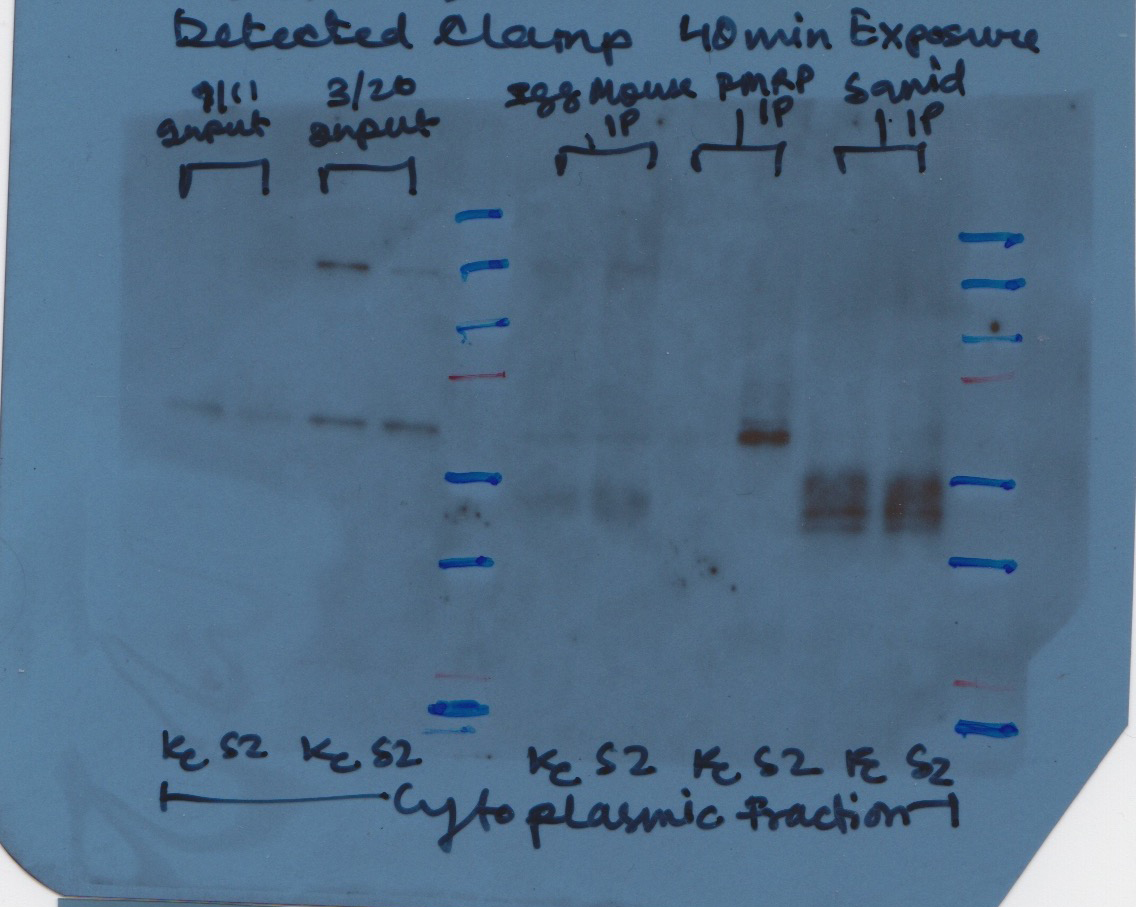

Supplement: Figure 5—source data 5. [file elife-87865-fig5-data5.zip › Figure 5E Source data 5_original]

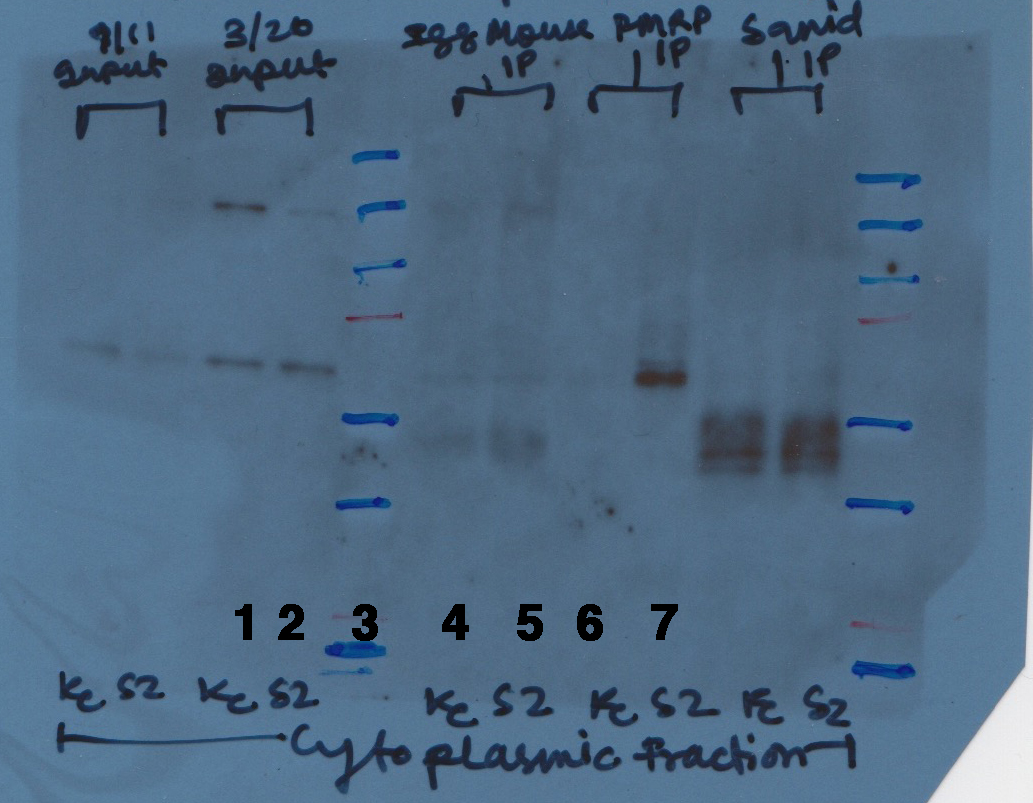

Supplement: Figure 5—source data 5. [file elife-87865-fig5-data5.zip › Figure 5E Source data 5_Cytoplasmic Fraction]

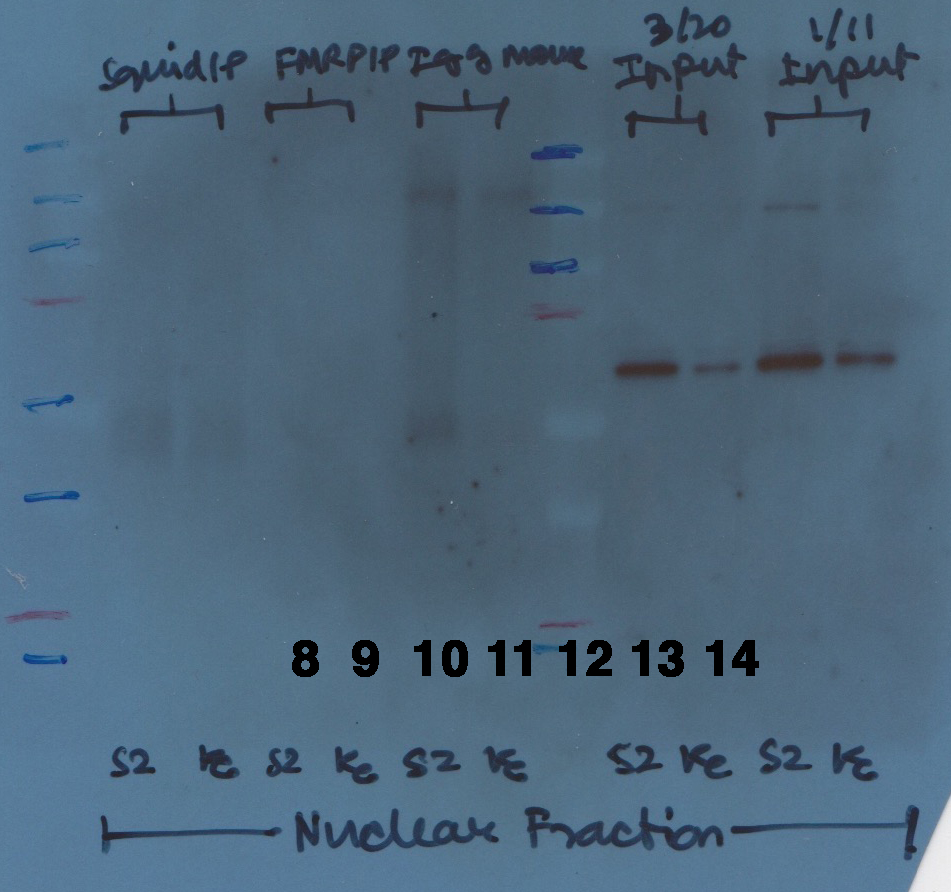

Supplement: Figure 5—source data 6. [file elife-87865-fig5-data6.zip › Figure 5E Source data 6_Nuclear Fraction]

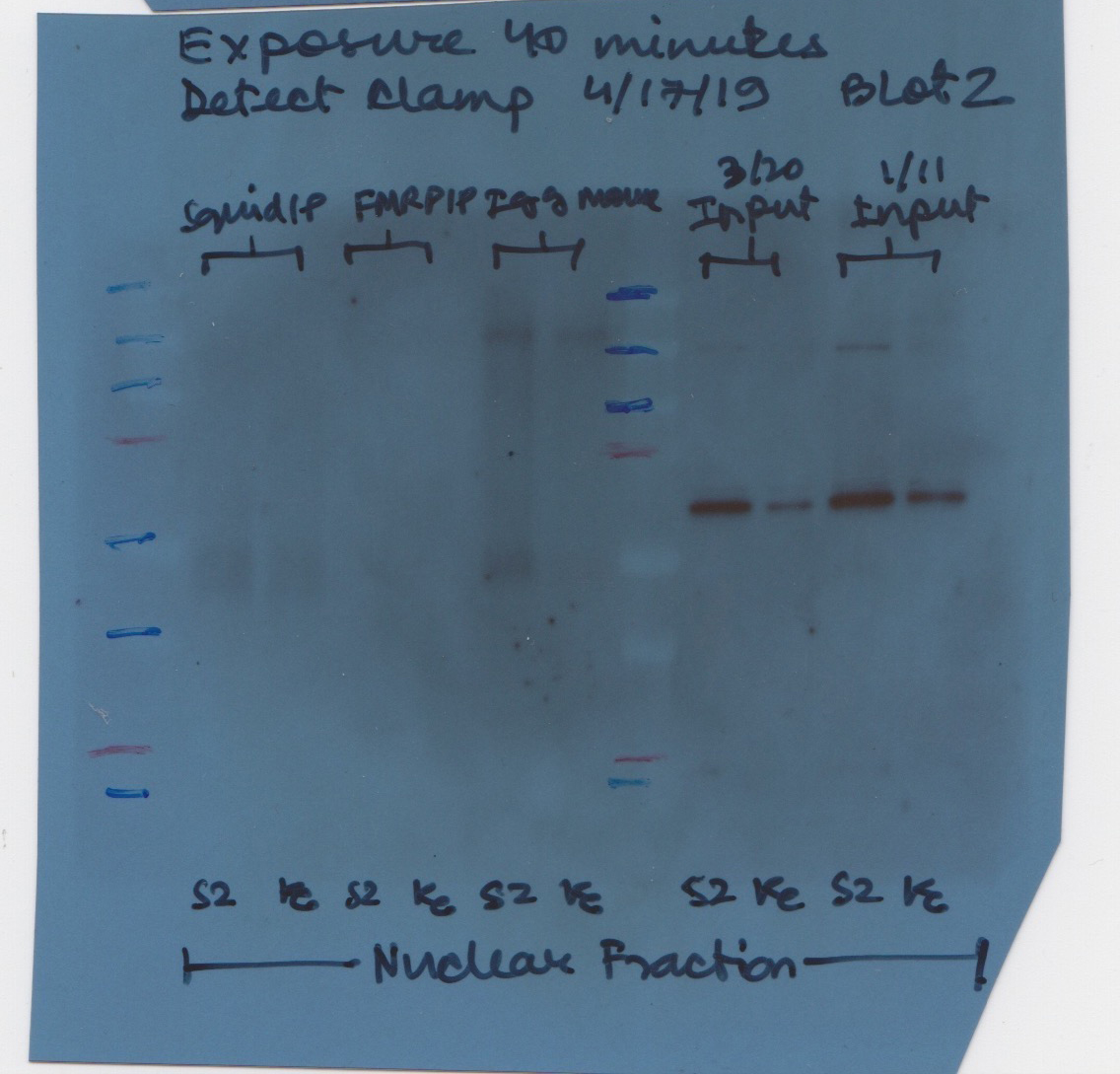

Supplement: Figure 5—source data 6. [file elife-87865-fig5-data6.zip › Figure 5E Source data 6_original]

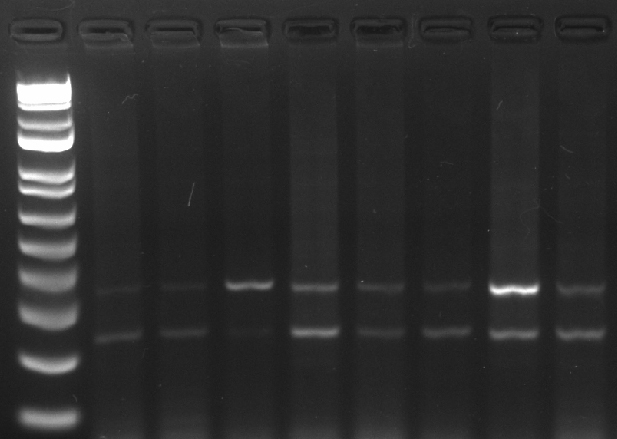

Supplement: Figure 6—source data 1. [file elife-87865-fig6-data1.zip › Figure 6B source data 1_original]

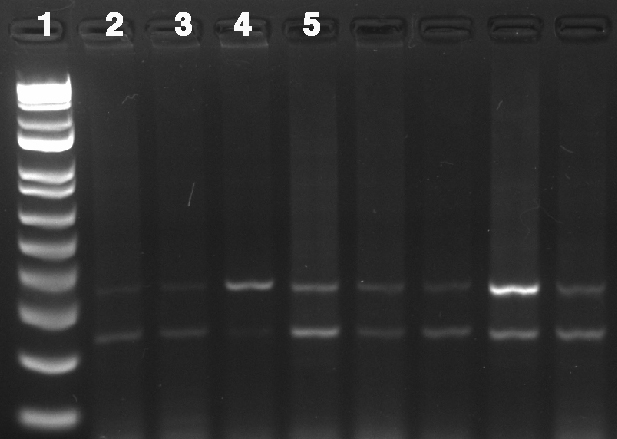

Supplement: Figure 6—source data 1. [file elife-87865-fig6-data1.zip › Figure 6B Source data 1_dsxF]

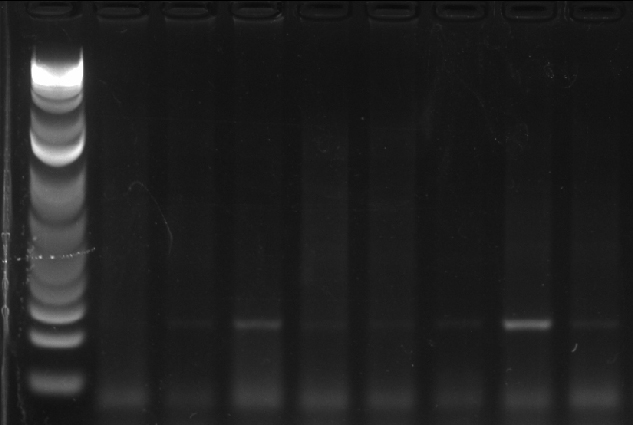

Supplement: Figure 6—source data 2. [file elife-87865-fig6-data2.zip › Figure 6B source data 2_original]

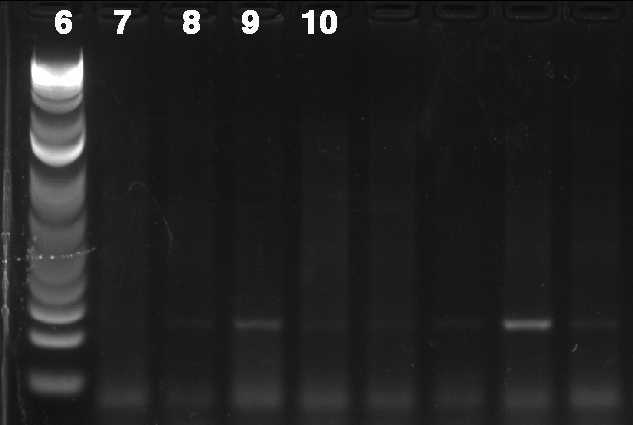

Supplement: Figure 6—source data 2. [file elife-87865-fig6-data2.zip › Figure 6B Source data 2_dsxM]

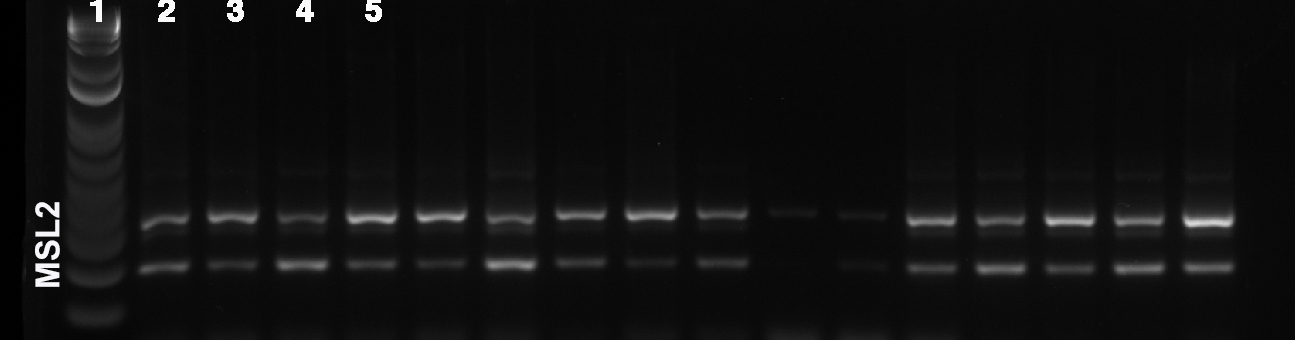

Supplement: Figure 6—source data 3. [file elife-87865-fig6-data3.zip › Figure 6C Source data 3_msl2]

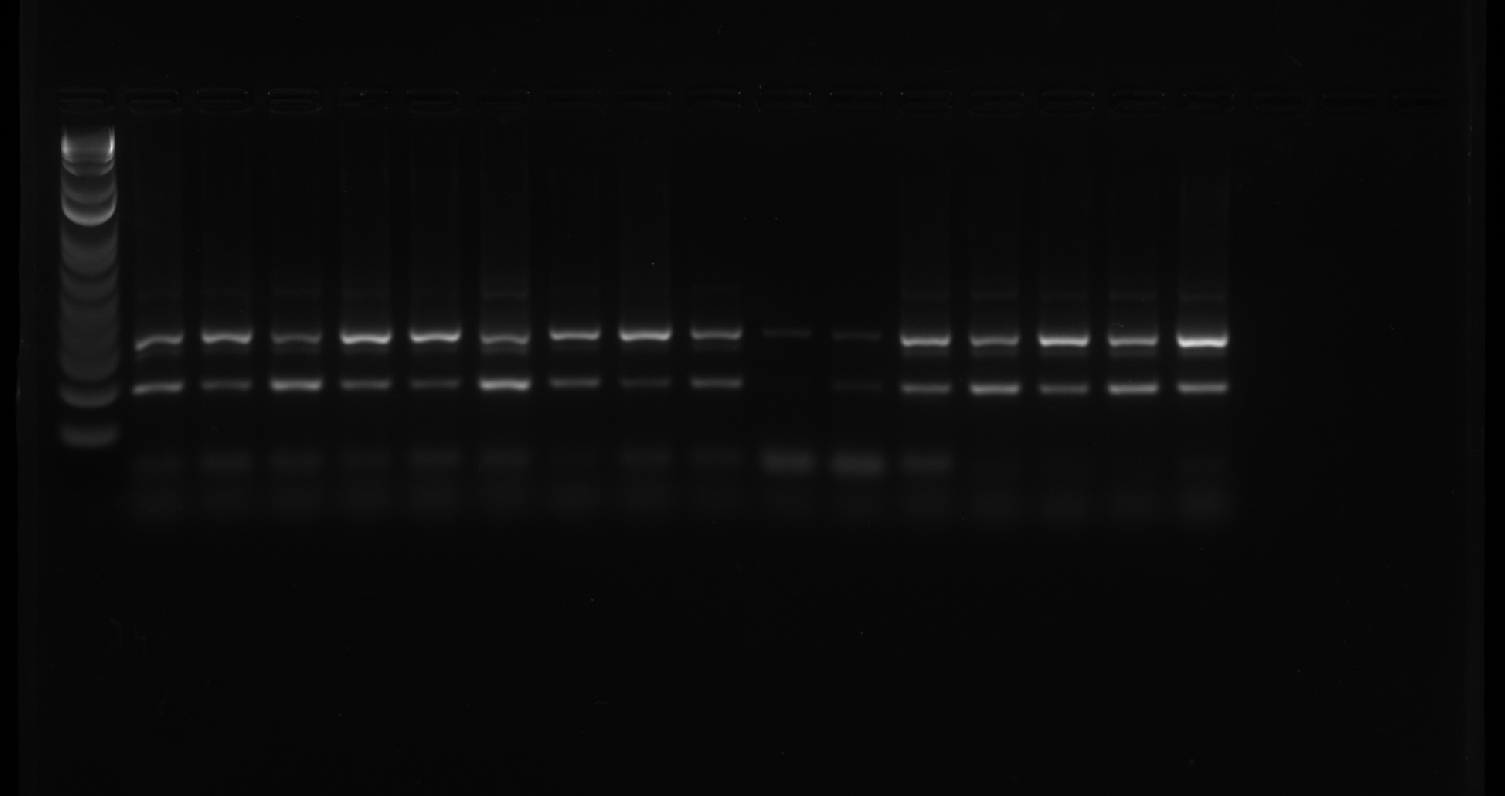

Supplement: Figure 6—source data 3. [file elife-87865-fig6-data3.zip › Figure 6C Source data 3_original]

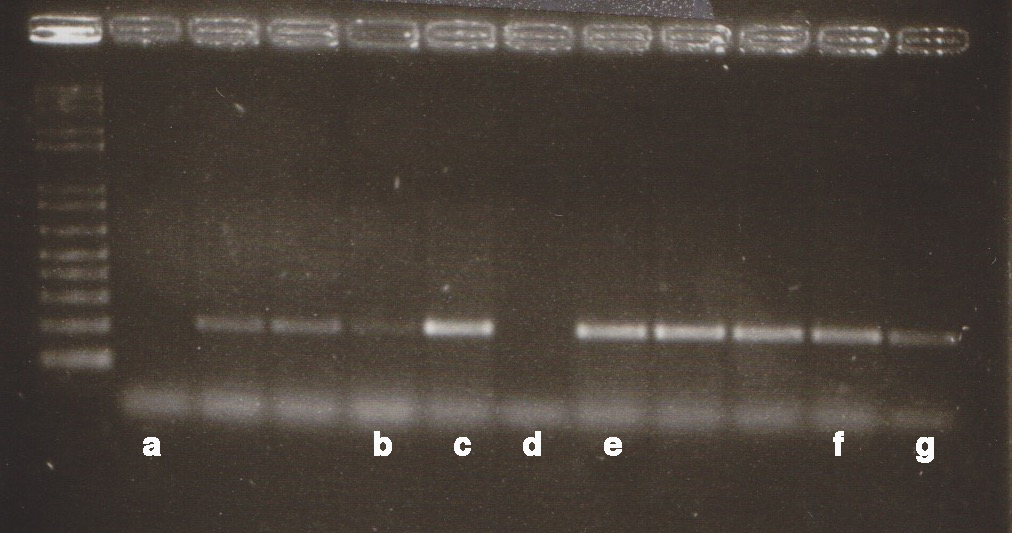

Supplement: Figure 6—source data 5. [file elife-87865-fig6-data5.zip › Figure 6D Source data 5_L3 male]

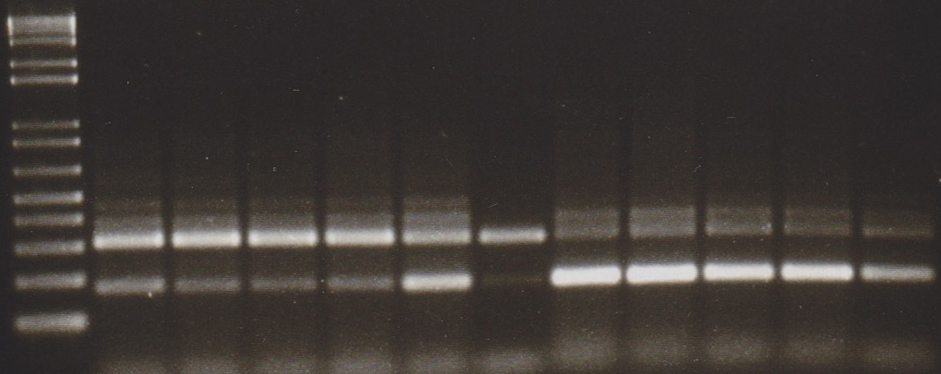

Supplement: Figure 6—source data 6. [file elife-87865-fig6-data6.zip › Figure 6E Source data 6_original]

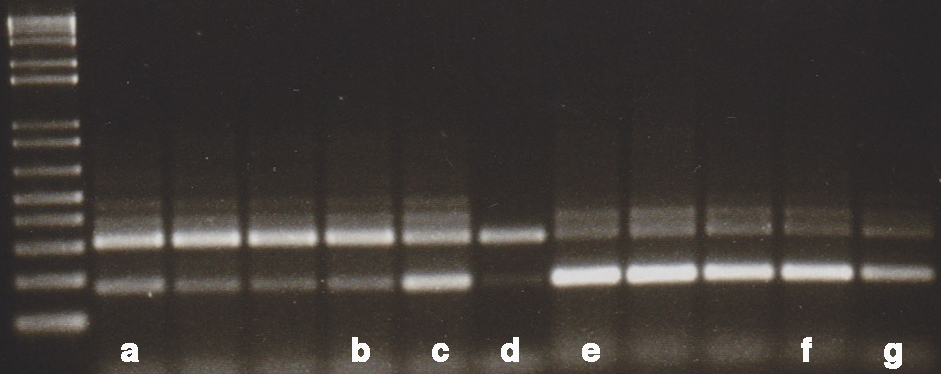

Supplement: Figure 6—source data 6. [file elife-87865-fig6-data6.zip › Figure 6E Source data 6]
