# Supplementary material for: Sex-specific splicing occurs genome-wide during early Drosophila embryogenesis
Source: eLife. 2023 Jul 19;12:e87865. doi: 10.7554/eLife.87865 (PMC10400075; doi:10.7554/eLife.87865)
Supplement: Supplementary file 2. [file elife-87865-supp2.docx]

**Supplementary File 2:** Summarizing the results and functions of the validated target genes (not part of sex-determination pathway) at which splicing is regulated by CLAMP

| **Gene name** | **Function** | **Effect on splicing** |
| --- | --- | --- |
| Fus (Fusilli) | Fus regulates alternative splicing of specific genes and plays a role in embryonic dorsoventral patterning (Wakabayashi-Ito 2001)^1^. Its human ortholog ESRP1 (Epithelial splicing regulatory protein) regulates splicing during the epithelial to mesenchymal transition and is implicated in autosomal recessive non-syndromic deafness 109. Alternative splicing of both Fus and ESRP1 has been shown to confer distinct subcellular localization (Yang and Carstens 2017)^2^. | CLAMP regulates splicing of retained intron in exon 97 of *fus* in males (**Figure 2-figure supplement 3A, D, G**). |
| Wnd (Wallenda) | encodes for a MAP Kinase with roles in axonal injury signaling and in regulation of presynaptic bouton structure (Russo et al 2019)^3^ | one of the isoforms isoA significantly downregulated only in males (**Figure 2-figure supplement 3B, E, H**) |
| PEP (Protein on ecdysone puffs) | PEP is part of the catalytic step 2 spliceosome (Herold et al 2009)^4^ and physically interacts with MLE (Cugusi et al 2015)^5^, Squid (Amero et al 1993)^26^, Ubx and Abd-A (Bischof et al 2018)^6^. | Splicing of intron between exon 6-5 in *pep* is regulated by CLAMP in males (**Figure 2-figure supplement 3E, J, O**). |
| spen | encodes an RRM (RNA recognition motif) domain protein that interacts with the *Hox* pathway (Willette et al 1999)^7^. It is orthologous to human SPEN (spen family transcriptional repressors) which recruits histone deacetylases. *de novo* truncating variants in *SPEN* have been linked to a neurodevelopmental disorder associated with obesity and increased BMI in females who also have a distinctive X chromosome epi-signature (Radio et al 2021)^8^. | *spen* exon5 skipped transcript is significantly upregulated in females (**Figure 2-figure supplement 3J, N**) and not in males |
| Ama (Amalgam) | regulates receptor ligand activity during cell-cell adhesion and positively regulates glial cell proliferation (Seeger et al. 1988, Fremion et al. 2000)^9^,^10^. Human ortholog LSAMP is implicated in ovarian and prostate cancer (Spears et al 2006, Petrovics et al 2015)^11,12^ | Isoform B show significant down-regulation in males after CLAMP RNAi (**Figure 2-figure supplement 3K, O**) compared to females |
| iab4 | non-coding RNA regulating *abd-A,* located within the essential *Hox* cluster that controls body plan patterning. CLAMP directly binds and regulates chromatin accessibility at this gene (Duan et al 2021)^13^. | retained intron isoform is significantly down-regulated in males in absence of CLAMP (**Figure 2-figure supplement 3L, P).** |
| sc35 (SR family splicing factor) | Sc35 regulates mRNA alternative splicing, the processing of mRNA 3’ends, and transcription start site selection. The human ortholog, SRSF2, is linked to acute myeloid leukemia and myelodysplastic syndrome in which females show a significant survival advantage over their male counterparts (Hossain and Xie 2015, Wang et al 2019)^14,15^. Affected men have overall more mutations in genes involved in RNA splicing and epigenetic regulation with a higher risk of disease progression and overall poor outcome (Karantanos et al 2021)^16^. | Splicing of a *sc35* isoform with exon7 is significantly affected in males and not females (**Figure 2-figure supplement 3M, Q**). |
| Bacc (Bacchus) | encodes for tyramine dependent nuclear regulators involved in ethanol sensitivity (Chen et al 2013)^17^. | CLAMP-dependent splicing in both males and females (**Figure 2-figure supplement 3M, R**), However, isoform B with exon3 is significantly down-regulated in males compared to females in absence of CLAMP. |

1. Wakabayashi-Ito N, Belvin MP, Bluestein DA, Anderson KV. Fusilli, an essential gene with a maternal role in Drosophila embryonic dorsal–ventral patterning. Developmental Biology 229, 44-54 (2001).

2. Yang Y, Carstens RP. Alternative splicing regulates distinct subcellular localization of Epithelial splicing regulatory protein 1 (Esrp1) isoforms. Scientific reports 7, 1-10 (2017).

3. Russo A, Goel P, Brace E, Buser C, Dickman D, DiAntonio A. The E3 ligase Highwire promotes synaptic transmission by targeting the NAD‐synthesizing enzyme dNmnat. EMBO reports 20, e46975 (2019).

4. Herold N, Will CL, Wolf E, Kastner B, Urlaub H, Lührmann R. Conservation of the protein composition and electron microscopy structure of Drosophila melanogaster and human spliceosomal complexes. Molecular and cellular biology 29, 281-301 (2009).

5. Cugusi S, Kallappagoudar S, Ling H, Lucchesi JC. The Drosophila helicase maleless (MLE) is implicated in functions distinct from its role in dosage compensation. Molecular & Cellular Proteomics 14, 1478-1488 (2015).

6. Bischof J, et al. Generation of a versatile BiFC ORFeome library for analyzing protein–protein interactions in live Drosophila. Elife 7, e38853 (2018).

7. Wiellette EL, Harding KW, Mace KA, Ronshaugen MR, Wang FY, McGinnis W. spen encodes an RNP motif protein that interacts with Hox pathways to repress the development of head-like sclerites in the Drosophila trunk. Development 126, 5373-5385 (1999).

8. Radio FC, et al. SPEN haploinsufficiency causes a neurodevelopmental disorder overlapping proximal 1p36 deletion syndrome with an episignature of X chromosomes in females. The American Journal of Human Genetics 108, 502-516 (2021).

9. Fremion F, Darboux I, Diano M, Hipeau‐Jacquotte R, Seeger M, Piovant M. Amalgam is a ligand for the transmembrane receptor neurotactin and is required for neurotactin‐medated cell adhesion and axon fasciculation in Drosophila. The EMBO Journal 19, 4463-4472 (2000).

10. Seeger MA, Haffley L, Kaufman TC. Characterization of amalgam: a member of the immunoglobulin superfamily from Drosophila. Cell 55, 589-600 (1988).

11. Spears M, et al. The function of tumor suppressor genes in ovarian cancer: the role of LSAMP.). AACR (2006).

12. Petrovics G, et al. A novel genomic alteration of LSAMP associates with aggressive prostate cancer in African American men. EBioMedicine 2, 1957-1964 (2015).

13. Duan JE, et al. CLAMP and Zelda function together to promote Drosophila zygotic genome activation. elife 10, 2020.2007. 2015.205054 (2021).

14. Hossain MJ, Xie L. Sex disparity in childhood and young adult acute myeloid leukemia (AML) survival: Evidence from US population data. Cancer epidemiology 39, 892-900 (2015).

15. Wang F, Ni J, Wu L, Wang Y, He B, Yu D. Gender disparity in the survival of patients with primary myelodysplastic syndrome. Journal of Cancer 10, 1325 (2019).

16. Karantanos T, Jain T, Moliterno AR, Jones RJ, DeZern AE. Sex-Related Differences in Chronic Myeloid Neoplasms: From the Clinical Observation to the Underlying Biology. International journal of molecular sciences 22, 2595 (2021).

17. Chen J, Wang Y, Zhang Y, Shen P. Mutations in Bacchus reveal a tyramine-dependent nuclear regulator for acute ethanol sensitivity in Drosophila. Neuropharmacology 67, 25-31 (2013).
